# Model-Based Cost-Utility Analysis of Combined Low-Dose Computed Tomography Screening for Lung Cancer, Chronic Obstructive Pulmonary Disease, and Cardiovascular Disease

**DOI:** 10.1016/j.jtocrr.2025.100813

**Published:** 2025-02-19

**Authors:** Carina M. Behr, Maarten J. IJzerman, Michelle M.A. Kip, Harry J.M. Groen, Marjolein A. Heuvelmans, Maarten van den Berge, Pim van der Harst, Marleen Vonder, Rozemarijn Vliegenthart, Hendrik Koffijberg

**Affiliations:** a Health Technology and Services Research, TechMed Centre, University of Twente, Enschede, The Netherlands; b Erasmus School of Health Policy & Management, Erasmus University Rotterdam, Rotterdam, The Netherlands; c Department of Pulmonary Diseases and Tuberculosis, UMCG - University Medical Center Groningen, Groningen, The Netherlands; d Department of Epidemiology, University Medical Center of Groningen, University of Groningen, Groningen, The Netherlands; e Department of Pulmonary Diseases, University of Groningen, University Medical Center Groningen, Groningen, The Netherlands; f Groningen Research Institute for Asthma and COPD, University of Groningen, University Medical Center Groningen, Groningen, The Netherlands; g Department of Cardiology, University Medical Center Utrecht, Utrecht, The Netherlands; h Department of Radiology, Medical Imaging Center, University Medical Center Groningen, University of Groningen, Groningen, The Netherlands; i Machine Learning Lab, Data Science Center in Health (DASH), University Medical Center Groningen, University of Groningen, Groningen, The Netherlands

**Keywords:** Population-based screening, Low-dose computed tomography, Lung cancer, Chronic obstructive pulmonary disease, Cardiovascular disease

## Abstract

**Introduction:**

The conditional cost-effectiveness of low-dose computed tomography for lung cancer (LC) screening has been reported. Extending LC screening to chronic obstructive pulmonary disease (COPD) and cardiovascular disease (CVD), together with Big-3, could increase health benefits at marginal costs. This study aimed to estimate the cost-utility of Big-3 screening compared with no screening and LC screening in The Netherlands.

**Methods:**

A microsimulation model was built to reflect the care pathway, using individual-level data from the National Lung Screening Trial individual-level data, and aggregated data from the literature. The model includes a simulation of the detection of the Big-3 diseases through screening and standard of care. The model also simulated tumor growth and the effects of smoking cessation and treatment. Hypothetical (former) smokers (aged 55–74 y) were simulated according to the National Lung Screening Trial criteria. Individuals with screening-detected diseases receiving (preventative) treatment experience a reduced risk of events and increased survival. A Dutch health system perspective and lifetime horizon were adopted.

**Results:**

Simultaneous LC and CVD screening was the most cost-effective, with incremental costs and effects of €1937 and 0.22 quality-adjusted life-years (QALYs) versus no screening, and €595 and 0.08 QALYs versus LC screening, respectively. This yielded incremental cost-utility ratios of €8561 per QALY and €7154 per QALY versus no screening and LC screening, respectively. LC plus COPD screening was dominated by LC + CVD screening, which yielded lower health benefits and higher costs.

**Conclusions:**

Simultaneous screening for LC + CVD in a high-risk population offers health benefits at low costs compared with no screening or LC screening alone. Adding COPD screening cannot yet be justified owing to the limited clinical evidence.

## Introduction

Lung cancer (LC), chronic obstructive pulmonary disease (COPD), and cardiovascular disease (CVD) are major causes of morbidity and mortality worldwide and are named the “Big-3 killers.”^1^ LC is the leading cause of cancer-related mortality, and COPD is the third leading cause of noncommunicable disease deaths globally.^2^ CVD is responsible for the highest number of deaths globally and is a major cause of disability and impaired quality of life.^3^ These Big-3 diseases can all be detected using a single low-dose computed tomography (LDCT) scan, through the presence of pulmonary nodules, emphysema or bronchial wall thickening, and coronary calcium scoring, which is a subclinical marker of atherosclerotic CVD (ASCVD).^4^ Furthermore, these diseases have smoking and aging as overlapping risk factors. As LDCT LC screening in high-risk individuals is gaining traction, additional screening for COPD or CVD may increase health benefits at additional marginal costs.

LDCT screening has been shown to reduce LC mortality by detecting LC at an early stage, thereby allowing curative treatment. The National Lung Screening Trial (NLST) reported that LDCT screening reduces LC mortality by 20% and all-cause mortality by 7%.^5^ In the Nederlands–Leuvens Longkanker Screenings Onderzoek (NELSON) trial, LC mortality was reduced by 25% although no reduction in all-cause mortality was shown.^6^ National screening trials in the United Kingdom, Germany, and other countries also reported LC-specific mortality benefits.^7^^,^^8^

Several studies have also investigated the cost-effectiveness of LDCT screening for LC on the basis of the benefits shown in clinical trials, and the majority have shown screening to be cost-effective under certain conditions.^9^ In these cost-effectiveness studies, the intervention (screening) and the comparator (no screening) were both modeled with all healthcare costs related to the diseases modeled (e.g., preventative treatment, diagnostic costs, and treatment costs) to make a direct comparison of the health effects and all costs to the health care system.

On the basis of mortality reduction and cost-effectiveness studies, several countries are advising or considering national LC screening programs for high-risk individuals. The U.S.Preventative Task Force recommends screening for current and former smokers aged 50 to 80 years with 20 or more pack-years of smoking history.^10^ Nevertheless, many European countries are still cautious and exploring which screening program setup (e.g., screening population and frequency) would be most cost-effective. “A New Approach” to cancer screening in the European Union recommends that countries investigate the feasibility of LC screening and start implementation trials.^11^

Including Big-3 screening is another avenue being explored to increase efficiency, as multiple diseases can be detected using a single scan. Nevertheless, few studies have investigated the health benefits of Big-3 combination screening. Several studies have investigated these diseases. One example is the Risk or Benefit in Screening for Cardiovascular Disease trial, which investigates the effect of coronary calcium scoring–based management.^12^ High calcium scores have been shown to have a strong association with CVD mortality and chronic heart disease (CHD) risk.^13^, ^14^, ^15^

To the best of our knowledge, health economic studies of Big-3 screening have not yet been performed.^9^ Initial work done by our group has reported that there is a health-economic argument, particularly for adding CVD to LC screening.^16^ This study did not provide the evidence but instead prioritized further research. This further research can be conducted from the perspective of the current standard of care is not only no screening but also LC screening, as the implementation of LC screening is possible in the near future. More advanced studies are limited by the lack of data on the effectiveness of screening for COPD and ASCVD using LDCT. Therefore, a full economic evaluation must be model-based, synthesizing existing data sources. Furthermore, investigating the cost-utility of Big-3 screening is challenging because it depends on contextual factors, such as the prevalence of COPD and CVD in the screening population and the consequences of (preventative) treatment for COPD and CVD.^16^

Therefore, this study aimed to develop a flexible model to estimate the cost-utility of Big-3 screening compared with no screening and LC screening in the Netherlands, which allows for future adjustments to emerging data.

## Materials and Methods

An individual-level state transition microsimulation model was developed using data from the NLST to estimate the long-term cost-utility of annual screening between the ages of 55 and 74 years for LC, LC plus COPD, LC + CVD, Big-3, and no screening (current standard). The model applies monthly cycles, reflecting the care pathways of hypothetical individuals, including the detection of the Big-3 diseases through screening or after experiencing COPD exacerbations or CVD events. The model also reflects tumor growth, the potential impact of smoking cessation, and the treatment of diagnosed diseases. The model was built using the DARTH framework^17^ in R version 4.2.2.^18^ Costs were discounted by 4.0% and effects by 1.5% according to Dutch guidelines,^19^ and the analysis applied a health system perspective and lifetime horizon. This study followed the Consolidated Health Economic Evaluation Reporting Standards Checklist.^20^

A flexible model was built to allow future adjustments to emerging data and support future research questions. Therefore, the model can evaluate screening benefits for different target populations, different combinations of LC, COPD, and CVD, and different screening intervals. The model simulated current and former smokers defined by the NLST inclusion criteria, aged 55 to 74 years, with more than 30 pack-years, who still smoked or quit smoking less than 15 years ago. Patients with screen-detected LC could receive early treatment owing to a stage shift (detection through screening occurs in earlier stages than detection without screening); patients with COPD experience a reduction in exacerbations and COPD-related mortality risk if they receive treatment (with beta-agonists); and individuals with an elevated ASCVD risk receive preventative treatment (with angiotensin-converting–enzyme inhibitors or statins), resulting in reduced ASCVD risk.

**Figure 1:**
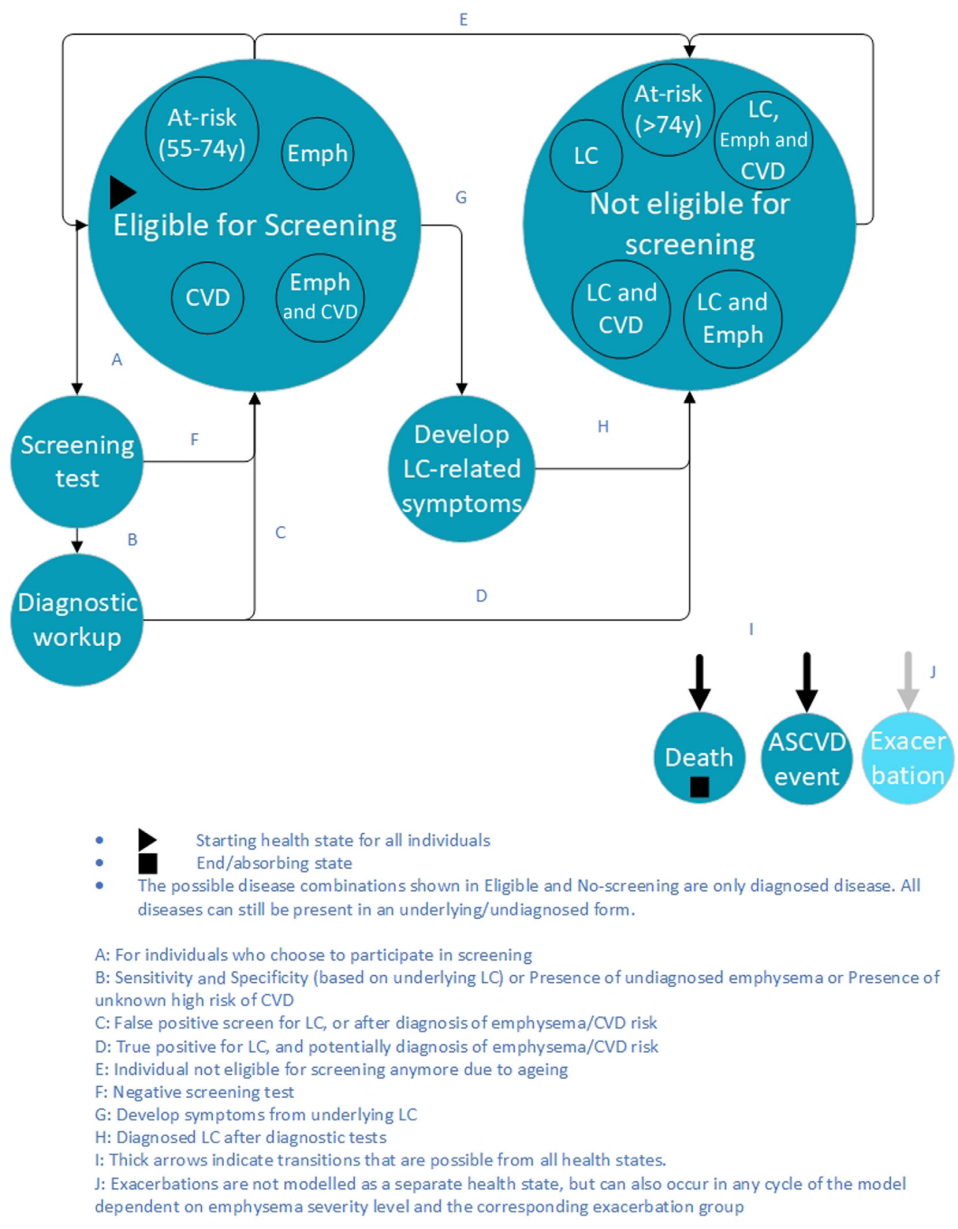
Model health states with possible transitions. ASCVD, atherosclerotic CVD; CVD, cardiovascular disease; Emph, emphysema; LC, lung cancer.

The health states of the model are shown in Figure 1, in which individuals can die or experience ASCVD events from any state in the model. All individuals start in the “Eligible for screening” health state. The diseases indicated as “Eligible for screening” and “Not eligible for screening” reveal which combinations of diagnosed diseases can be experienced by individuals in these states. Undiagnosed diseases are not indicated in the figure as they are the underlying individual characteristics in the model. Individuals who are in the state “Eligible for screening,” can have combinations of COPD or elevated CVD-risk, but cannot have diagnosed LC. Individuals diagnosed with LC are no longer eligible for screening. Regarding screening strategies, individuals decide to participate in a screening program. If so, the individual transitions to the “Screening test” state, in which the LDCT screening is conducted. After a negative scan, individuals return to “Eligible for screening.” After a positive screen, individuals move to “Diagnostic workup” and subsequently to “Not eligible for screening” if LC is diagnosed or “Eligible for screening” if no LC is diagnosed. Emphysema and CVD risk will also be evaluated or identified at the baseline screen only, which affects costs and health effects but not the state to which individuals move. Individuals “eligible for screening” can develop LC symptoms between screening rounds for an undiagnosed tumor, either newly formed or missed during previous screening rounds. When such symptoms occur individuals will seek medical care, a diagnosis will be made and these individuals move to “Not eligible for screening.” In any health state, a person with emphysema can experience COPD exacerbation (which is not modeled as a health state but as an underlying individual characteristic). Individuals who reach 74 years of age, move from “Eligible for screening” to “Not eligible for screening.” The model code and detailed description of the model are available online at github.com/Carinitha/B3CareModel.

Individual-level data from the NLST were used to develop the model. Therefore, this analysis used the same inclusion criteria for smoking history and an age requirement of 55 to 74 years. In every simulation run, 10,000 individuals were sampled from the NLST population data; that is, the initial age, sex, smoking status (current or former smoker), number of pack-years smoked, family history of LC, CVD, COPD, or emphysema history of individuals from the NLST data set. Thereafter, every individual is assigned an underlying centrilobular emphysema severity level (Fleischner classification) on the basis of aggregate data obtained from the Imaging in Lifelines study.^1^^,^^21^ Each individual is also assigned an underlying risk of CHD and stroke, which are the two CVD risks included in this model, as reflected by the coronary artery calcium scores. Individual CHD risk was calculated according to the Multi-Ethnic Study of Atherosclerosis risk prediction model,^22^ to be able to capture the effect of calcium scores on the individual-level risk of CHD. This risk-prediction model was based on the assumption that the entire population was Caucasian and had a distribution of blood pressure and cholesterol levels in the LC screening-eligible subpopulation of the Multi-Ethnic Study of Atherosclerosis trial. Each individual’s risk of experiencing a stroke was calculated using the Framingham stroke risk equation (excluding calcium scores)^23^ and the risk of those receiving treatment was adjusted for relative risk. After screening, individuals with moderate or severe emphysema were sent for COPD evaluation during outpatient visits, including spirometry testing, to inform about treatment to limit exacerbations and symptoms. Individuals with moderate or severe coronary calcium, apparent on screening, were sent to a general practitioner and assigned preventative treatment on the basis of the proportion of individuals receiving treatment in the ROBINSCA trial. For example, 22% of individuals who do not have a history of CVD and are classified as being at increased risk receive angiotensin-converting–enzyme inhibitors inhibitors.^24^

### Input Parameters

The input parameters (Table 1^1^^,^^21^^,^^22^^,^^25^, ^26^, ^27^, ^28^, ^29^, ^30^, ^31^, ^32^, ^33^, ^34^, ^35^, ^36^, ^37^, ^38^, ^39^, ^40^, ^41^, ^42^, ^43^, ^44^, ^45^, ^46^, ^47^, ^48^, ^49^, ^50^, ^51^) were derived from NLST data and gathered from the literature. The parameter dependence on the individual characteristics is indicated in the table; nevertheless, aggregated values are reported. For more details, refer to the online model in the Supplementary Material. Every individual in the model was assigned a probability of attending the screening, the probability of dying of causes other than the Big-3 on the basis of age and sex, and the probability of dying of one of the Big-3 diseases on the basis of age, sex, LC stage, COPD severity, and CVD risk. Individuals without LC are at risk of developing LC on the basis of the PLCOm2012 risk model,^26^ converted to risk per cycle. When a new tumor develops in the model, it has a certain starting diameter, a volume doubling time, and a size at which it will be detected without screening, as described in the Calibration section. Every individual with emphysema was categorized into an exacerbation group (on the basis of the presence of centrilobular emphysema among current and former smokers in the Imaging in Lifelines study^1^), determining the rate of exacerbations (0–4 per y).

**Table 1:** Input Parameters With the Values, Source, Country the Data is From and the Population the Data is Based On

Parameter	Value (Mean ± SD)	Country	Population	Distribution	Source
Transition probabilities					
Probability of attending screening	0.75, Range: 0.63–0.97 4 beta regression models, depending on which disease(s) are being screened for in the modeled strategy	International	Current and former smokers	Beta	^25^
6-y LC risk according to PLCOm2012 simplified risk model converted to monthly probabilities	Current smoker: 0.10068 ± 0.05 Former smoker: 0.06526 ± 0.05 on the basis of age and smoking status (current or former smoker) range: 0.006–0.323	United States	Current and former smokers with mean pack-y of the NLST population (40 cigarettes per d for 29 y)	Beta	^26^
Proportion of individuals stopping smoking	Uniform distributions: Without screening: 0.0 to 0.6 With screening: 0.1 to 0.6, but always larger than or equal to the proportion without screening				Assumption on the basis of expert opinion
Annual probability of dying of other causes	0.01 ± 0.005	United States	NLST	Beta	^27^
Monthly probability of dying of LC according to stage	Gompertz distribution parameters (See supplements for survival curves) IA: −0.025 to 0.007 IB: −0.020 to 0.007 IIA: −0.028 to 0.015 IIB: −0.046 to 0.016 IIIA: −0.033 to 0.034 IIIB: −0.038 to 0.051 IV: −0.075 to 0.143	United States	NLST	Gompertz	^27^
Cumulative LC incidence rate (lifetime)	0.22 ± 0.05	Netherlands	Heavy smokers	Beta	^28^
LC VDT, geometric mean log-transformed Geometric mean (d)	4.59 ± 0.21 98	United States	50–80-y-old current and former smokers	Lognormal	^28^
Self-detection tumor diameter	Mean = 45.9 mm Shape = 3.74 Rate = 0.08		Distribution of NLST, with the mean calibrated	Gamma	Calibrated
Starting diameter of tumor at first appearance	0.33 mm			Fixed	Calibration
LDCT screening sensitivity dependent on LC tumor diameter (d)	If d < 3mm = 0% If 3 mm ≤ d < 5 mm = (0.5 × d − 1.5) × 100% If d ≤ 5 mm = 100%	Netherlands	Artificial pulmonary nodules	Beta	^28^
LDCT screening specificity for LC	99.2% ± 0.076%	Italy	Screening population	Beta	^29^
Presence of emphysema (these are aggregated values, which were further stratified on the basis of age categories in the model)	Men No emphysema: 34% Mild: 41% Moderate: 22% Severe: 1% Very severe: 2% Women No emphysema: 40% Mild: 51% Moderate: 7% Severe: 1% Very severe: 1%	Netherlands	Current and former smokers	Beta	^1^^,^^21^
Annual exacerbation rate per emphysema severity level	Mild: 0.754 Moderate: 0.865 Severe: 1.095 Very severe: 1.35 These are the average values, the more detailed values on the basis of exacerbation groups are available in the online model description.	United Kingdom	Current and former smokers with diagnosed COPD over the age of 40 y	Beta	^30^
Relative risk (reduction) of exacerbation with treatment	0.82 ± 0.0475		Pooled estimate	Beta	^31^
Relative risk (reduction) of exacerbation when one quits smoking	0.78 ± 0.03	United States	Current and former smokers	Beta	^32^
COPD mortality hazard ratio depending on the previous year’s exacerbations	0 exacerbations: reference 1–2 exacerbations: 2.00 (1.01–3.98) 3+ exacerbations: 4.13 (1.8–9.45)	Spain	Male patients with stable COPD	Truncated normal with cut-off values set at the 95% confidence interval and ensuring the 3+ value is always larger	^33^
CHD event risk	According to the MESA prediction model	United States	Individuals aged 45–84 y, free of clinical heart disease at baseline		^22^
Stroke risk	According to the Framingham stroke risk prediction model	United States and France	Based on three cohorts		^34^
Distribution of type of CHD events	Non-fatal myocardial infarction: 45% Angina: 35% CHD death: 16% Cardiac arrest: 4%	United States	Individuals aged 45–84 y, free of clinical heart disease at baseline	Dirichlet	^22^
Relative risk (reduction) with statin treatment	CHD: 0.62 ± 0.05 Stroke: 0.83 ± 0.04		Meta-analysis	Beta	^35^
Relative risk (reduction) with ACE inhibitor treatment	CHD: 0.79 ± 0.04 Stroke: 0.86 ± 0.05		Meta-analyses	Beta	^36^ ^37^
Costs (converted to 2022 euros)					
Invitation costs	6^a^	Netherlands	Colorectal cancer screening	Gamma	^38^
LDCT scan	176^a^	Netherlands	National healthcare products	Gamma	^39^
Diagnosis (PET scan and biopsy)	1747^a^	Netherlands	National healthcare products	Gamma	^39^
LC per stage	Once-off surgery cost: 15,840 Monthly costs per stage I and II: 60 ± 13 III: 710 ± 18 IV: 899 ± 106	Netherlands Australia	Retrospective national data	Gamma	^40^^,^^41^
LC treatment duration (mo)	I–III: 20.9 ± 26 IV: 12.6 ± 11	Netherlands	A random sample of Dutch hospital patients	Normal	^42^
COPD-related costs	Mild: 288 ± 293 Moderate: 358 ± 364 Severe: 339 ± 406 Very severe: 593 ± 602 Exacerbation: 525 ± 8,049	Netherlands	Patients with COPD	Gamma	^43^
Annual statin cost	40^a^	Netherlands	National healthcare products	Gamma	^44^
Annual ACE inhibitors cost	305^a^	Netherlands	National healthcare products	Gamma	^45^
CVD event costs:					
Myocardial infarction	6348 ± 8732	Netherlands	Inpatient and outpatient care after events	Gamma	^46^
Cardiac arrest and Angina	33,575 ± 19,612	Netherlands	Inpatient and outpatient care after events	Gamma	^47^
Stroke (monthly)	1–6 mo: 2936 ± 1532 6–13 mo: 780 ± 975	Netherlands	Inpatient and outpatient care after events	Gamma	^48^
Baseline utilities					
Baseline	Age ≤ 60 y 0.857 ± 0.183 Age 60–70 y 0.839 ± 0.179 Age > 70 y 0.852 ± 0.148	Netherlands	Dutch EQ-5D-5L	Beta	^49^
Relative disutilities					
COPD overall	GOLD 1 0.90 ± 0.11 GOLD 2 0.76 ± 0.27 GOLD 3 0.75 ± 0.30 GOLD 4 0.55 ± 0.31	Netherlands	Patients with COPD	Beta	^43^
Exacerbation	Moderate: 0.98 Severe: 0.95	Netherlands	Patients with COPD		^43^
LC per stage	I: 0.71 ± 0.005 II: 0.68 ± 0.010 III 0.67 ± 0.005 IV: 0.66 ± 0.005	United States	Patients with LC	Beta	^50^
CVD event (first month)	Stroke 0.64 ± 0.02 Coronary heart disease event 0.883 ± 0.01	United States	Self-reported in survey	Beta	^51^
CVD event (after the first month)	Stroke 0.80 ± 0.01 Coronary heart disease event 0.884 ± 0.01	United States	Self-reported in survey	Beta	^51^

ACE, angiotensin-converting-enzyme; CHD, chronic heart disease; COPD, chronic obstructive pulmonary disease; CVD, cardiovascular disease; LC, lung cancer; MESA, Multi-Ethnic Study of Atherosclerosis; NLST, National Lung Screening Trial; PET, positron emission tomography; PLCOm2012 LDCT, low-dose computed tomography; VDT, volume doubling time.

a For the values in which SDs were not available, the SD was set to 20% of the mean value.

The probability of screening attendance was determined using four beta regression models, accounting for the likelihood of individuals participating in LC, LC plus COPD, LC + CVD, or Big-3 screening respectively. The models were fitted to survey data from four European countries, completed by over 1200 current and former smokers.^25^ The models were used at the individual level, with predictors being age, sex, smoking status (current or former), family history of LC, diagnosis of COPD or CVD before screening, smoking history (pack-y), interaction factors between smoking status and diseases present, and between age and pack-years. These prediction models for screening different disease combinations were independent of each other although the same predictors were used. This means that, in the simulation model, it is possible for an individual to participate in LC screening but not in Big-3 screening if the combination of individual characteristics results in a lower willingness to participate in screening for Big-3 than LC alone. If an individual participates in screening, they also have a higher probability of smoking cessation than when there is no screening offered or they choose not to participate in screening. The probability of smoking cessation was modeled independently of the screening result as there is no consensus on whether smoking cessation rates are higher after a positive screening result.^52^

### Calibration

The starting diameter of lung tumors (the first moment that a lung nodule seems and is undetected in the model) and the mean diameter of a lung tumor at detection were calibrated to the expected proportion of the screening population with LCs at the first round of screening and to the expected diagnosed LC stage distribution with and without LC screening from the NELSON trial after 10 years and three screening rounds.^53^ This was done when considering that the starting diameter should be 3 mm or smaller, as this is the smallest screen-detectable size. A new tumor grows according to the assigned volume doubling time, which varies across individuals, as described by Du et al.^28^ Furthermore, the distribution of the tumor diameter at detection without screening or between screening rounds was calculated from the radiograph group (control group) of the NLST, after which the mean and SD of this distribution were calibrated to be a truncated gamma distribution with a maximum value of 40 mm to ensure that the resulting mean was realistic.

The model starts with all individuals in the “Eligible for screening” state with a warm-up time, that is, before screening starts, to already have some individuals with underlying tumors at the first screening round. The tumors that developed during the warm-up time which were already large enough to be detected, were removed from the model so that only undetected tumors remained at the first screening round. The number of undetected LCs developed before the first screening round could take place was calibrated to the LC detection rate in the NELSON trial after one screening round.^54^

### Validation

Throughout model development and up to the final analysis, three face-to-face validation meetings with experts (pulmonologists [HG, MH, MB], technical physicians [MV], radiologists [RV], and cardiologists [PH]) were organized to validate the model, including inputs, interim outcomes, and results. The model was also validated by comparing interim results with published results from the NELSON and NLST trials and publicly available data on CVD and COPD in the Netherlands (number of screen-detected LC, the prevalence of COPD and CVD in smokers, nodule size distribution, and tumor stage). Interim results that were compared are the stage distribution of LC at diagnosis for no-screening and LC screening and the proportion of the screening population with diagnosed LC compared with the 10-year results from NLST.^53^ Furthermore, the validation checklist from the Assessment of the Validation Status of Health-Economic Decision Models (AdViSHE) was applied.^55^ The checklist and results that were compared with the literature can be found in the Supplementary Material.

### Analysis

The model estimated the incremental cost-utility ratio (ICUR) of Big-3 screening in a fully incremental analysis of combinations of screening with LC and no screening, with costs expressed in 2022 Euros and effects expressed as quality-adjusted life-years (QALYs). The interim outcomes included the number of patients diagnosed with each disease (per stage) with and without screening, the number of false positives, and absolute mortality related to each disease.

All input parameters were defined by distributions, and for costs for which no data to inform a distribution were available, we assumed a 20% increase or decrease in the average cost. The percentage of individuals who stopped smoking after screening varied using a uniform distribution between 0 and 60% on the basis of the literature and expert opinions. A probabilistic analysis was performed to reflect the parameter uncertainty using 1000 simulation runs. This analysis provides insight into the overall uncertainty of the results on the basis of all the input parameters and does not focus on measuring the influence of the uncertainty of specific input parameters.

### Willingness-to-Pay

Because Big-3 screening focuses on multiple diseases with different severities and, therefore, potentially different willingness-to-pay (WTP) thresholds, all three WTP thresholds typically used in the Netherlands (i.e., 20, 50, and 80 €/QALY) were applied to inform policymakers.^19^

## Results

Simulating 10,000 individuals in a deterministic analysis resulted in intermediate outcomes, as shown in Table 2. Owing to the interplay between diseases, some results might differ from the initial expectations. For example, the number of individuals dying of LC in LC plus COPD screening is higher than when screening only for LC, whereas the total number of deaths decreases after 10 years. This is due to some individuals dying of causes other than LC (e.g., COPD) during LC screening, whereas during LC plus COPD screening, fewer individuals die from COPD and therefore more die of LC at a later time, but still within 10 years. Regarding emphysema-related results, the number of exacerbations declined when implementing LC screening because some individuals stopped smoking, resulting in a reduced risk of exacerbations. The number of diagnoses over a lifetime is lower than the number of underlying COPD cases because individuals who do not experience exacerbations do not attend screening or die before being diagnosed. It is also possible that individuals who experience exacerbations remain undiagnosed.

**Table 2:** Intermediate Outcomes for 10,000 Individuals in the Screening Population

	No Screening	LC Screening	LC + COPD Screening	LC + CVD Screening	Big-3 Screening
General					
Total mortality in 10 y	1270	1180	1169	1159	1142
LC	371	283	285	282	279
COPD	130	129	124	129	125
CVD	258	257	256	236	232
Other	511	511	504	512	506
Emphysema-related					
No. of moderate exacerbations over a lifetime	145,158	144,116	138,032	144,101	137,646
No. of severe exacerbations over a lifetime	12,428	12,405	11,809	12,405	11,791
No. exacerbations in y 1	4631	4612	4138	4612	4112
No. COPD diagnoses in y 1	1592	1591	4235	1591	4393
No. of COPD diagnoses over a lifetime	1792	1790	4307	1791	4455
Total no. of COPD cases	5535	5535	5535	5535	5535
No. of participants who quit smoking	542	898	895	898	904
No. of individuals who smoke in the starting population	4867	4867	4867	4867	4867
CVD-related (after 10 y)					
No. of MIs	10,495	10,492	10,496	10,449	10,453
No. of cardiac arrests	10,040	10,039	10,039	10,036	10,036
No. of angina	989	1002	996	952	953
No. of strokes	10,549	10,561	10,563	10,526	10,525
No. of individuals getting statins	743	743	743	2053	2084
No. of individuals getting ACE inhibitors	1426	1426	1426	2261	2276
No. of individuals dying of CHD event	183	181	179	166	162
No. of individuals dying of ASCVD event	258	257	256	236	232
LC-related					
No. of LC diagnoses	2117	2250	2252	2274	2279
No. of LC cases over lifetime	4895	4865	4878	4897	4908
LC Stage distribution (after 10 y), %					
Stage I	37.5	70.3	69.8	70.7	71.1
Stage II	8.8	6.3	6.3	6.1	6.1
Stage III	19.1	11.3	11.4	11.3	10.9
Stage IV	34.7	12.2	12.4	11.9	11.9

ACE, angiotensin-converting-enzyme; ASCVD, atherosclerotic cardiovascular disease; CHD, coronary heart disease; COPD, chronic obstructive pulmonary disease; CVD, cardiovascular disease; LC, lung cancer; MI, myocardial infarction.

The corresponding total and incremental costs and effects for all strategies considered are listed in Table 3. The results are presented as the total cost and effects per individual in the screening population. Screening for LC only compared with no screening resulted in incremental costs and effects of €1342 and 0.143 QALYs, and an ICUR of €9378 per QALY. The addition of COPD or CVD improved health outcomes and increased costs compared with LC screening, with the addition of CVD resulting in a more favorable ICUR because of a larger improvement in health outcomes and a smaller increase in costs compared with the addition of COPD to LC screening. The ICUR of LC + CVD compared with no screening was €8561 per QALY, and that of LC screening was €7154 per QALY, on the basis of incremental costs and effects of €595 and 0.083 QALYs, respectively. The ICUR of Big-3 screening is very large (€137,690 per QALY) because of the small incremental effect of additional COPD screening on the additional cost compared with LC + CVD screening. Therefore, screening for LC + CVD is the most cost-effective strategy for this model analysis. Both LC + CVD and Big-3 screening reported promising results; nevertheless, LC + CVD screening is considered more cost-effective because the resulting ICUR value is lower than the lowest WTP threshold applied (€20,000 per QALY).

**Table 3:** Base Case Cost-Utility Results

Strategy	Total Cost (€)	Total Effect (QALY)	Incremental Cost (€)	Incremental Effect (QALY)	ICUR (€ per QALY)
No-screening	6337	14.902	-	-	-
LC screening	7679	15.045			Weakly dominated
LC + CVD screening	8274	15.128	1937	0.226	8561
LC + COPD screening	8796	15.048			Dominated
Big-3 screening	9482	15.137	1208	0.009	137,690

COPD, chronic obstructive pulmonary disease; CVD, cardiovascular disease; ICUR, incremental cost-utility ratio; LC, lung cancer; QALY, quality-adjusted life-year.

**Figure 2:**
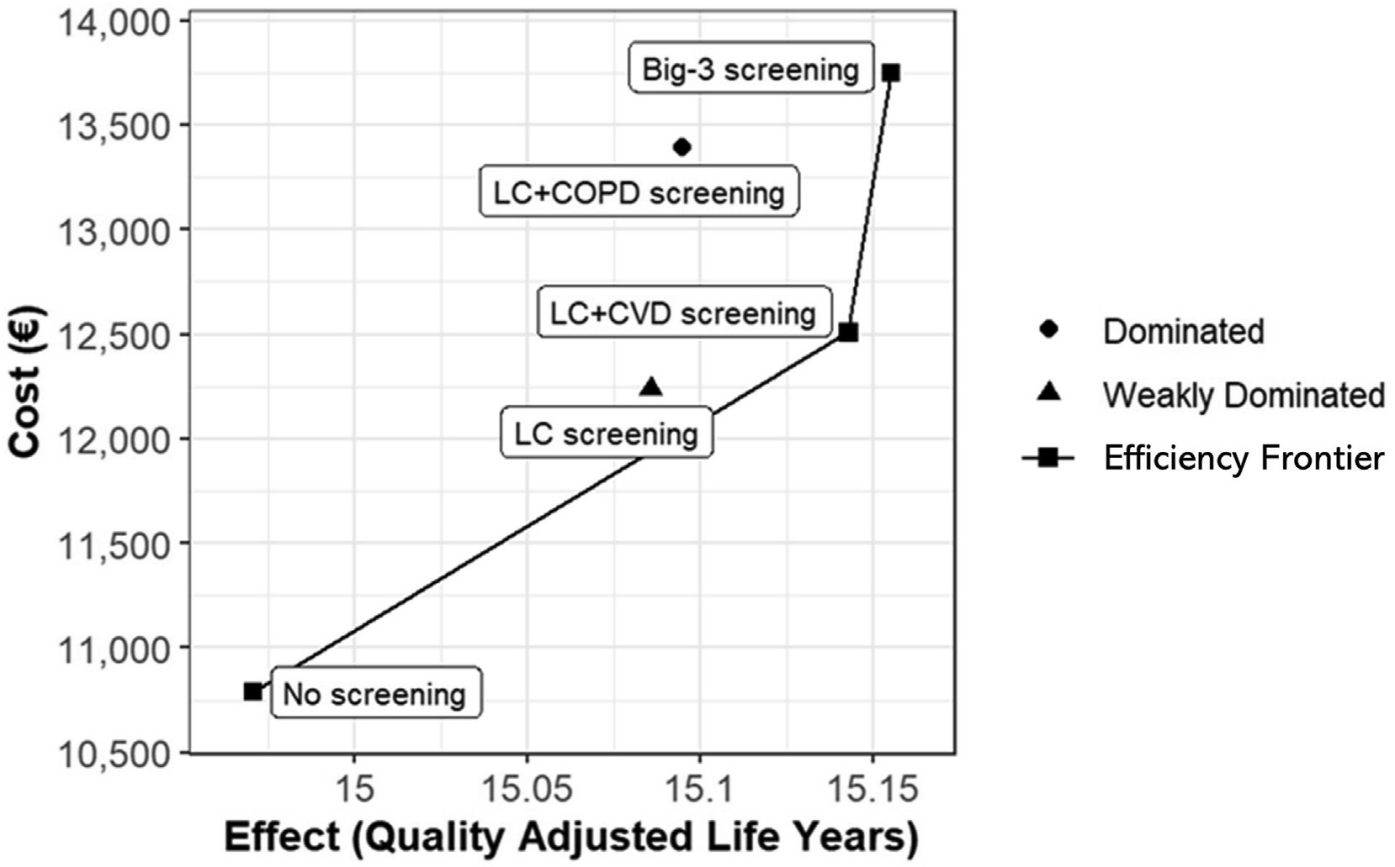
Cost-effectiveness plane. CVD, cardiovascular disease; COPD, chronic obstructive pulmonary disease; LC, lung cancer.

Figure 2 shows the efficiency frontier in the cost-effectiveness plane without uncertainty. From this figure, it can be observed that no screening, LC + CVD screening, and Big-3 screening lie on the efficiency frontier, although the steep efficiency frontier shows that the additional effect is limited compared with the additional cost. LC and LC plus COPD screenings were dominant. LC screening is weakly dominant, as it lies to the left of the efficiency frontier, and LC plus COPD screening is dominated by LC + CVD screening which is estimated to be both cheaper and more effective. It should be noted that the axes do not start at 0.

### Probabilistic Analysis

**Figure 3:**
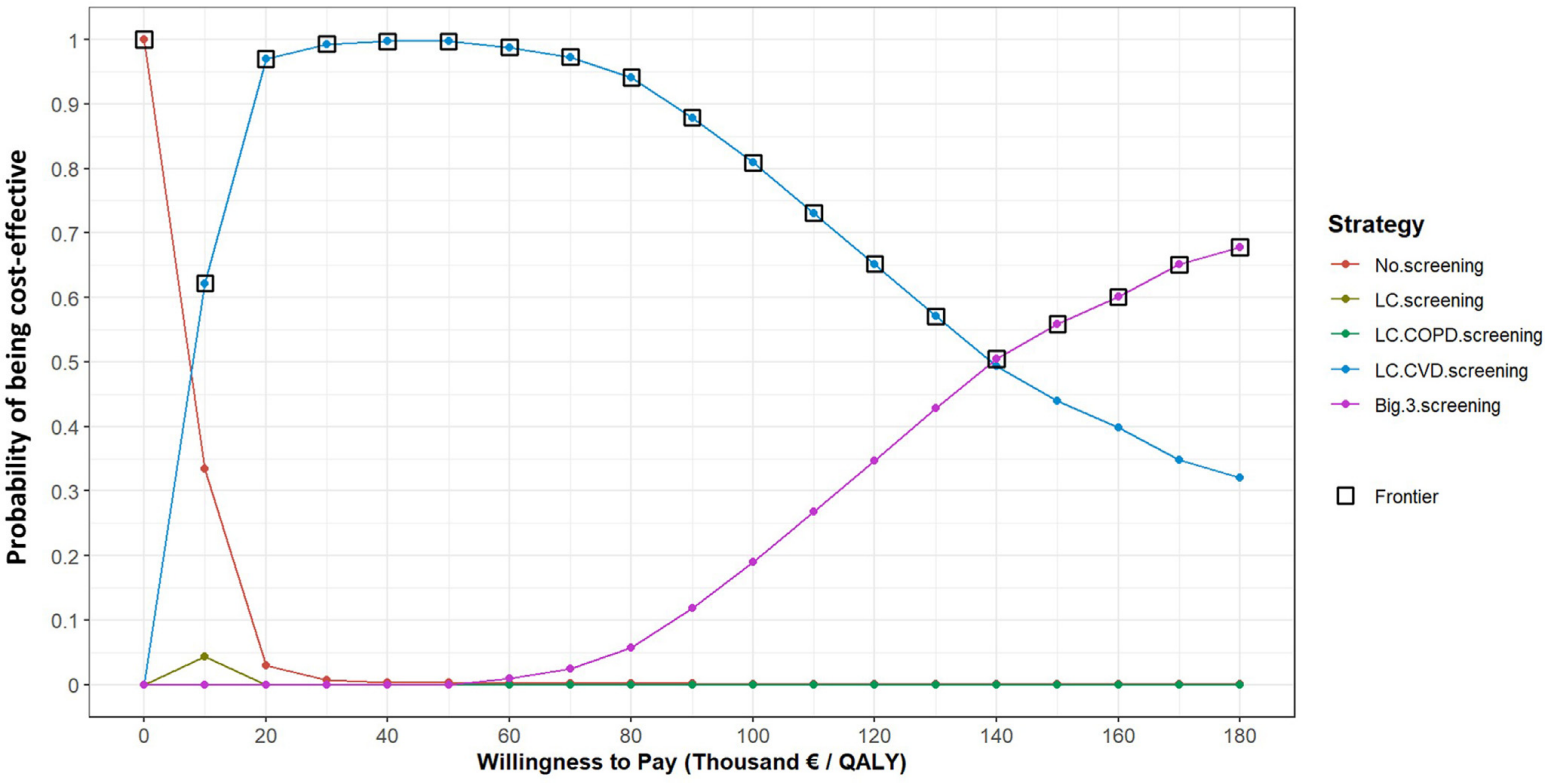
Cost-effectiveness acceptability curve indicating the probability of each screening strategy being cost-effective. CVD, cardiovascular disease; COPD, chronic obstructive pulmonary disease; LC, lung cancer; QALY, quality-adjusted life-year.

The cost-effectiveness acceptability curve in Figure 3 shows that the cost-effectiveness of LC + CVD screening was 97.0% at a WTP threshold of €20,000 per QALY, increasing to 99.7% at €50,000 per QALY and then declining to 94.1% at €80,000 per QALY. From a WTP of €135,000 per QALY, the Big-3 screening had the highest chance of being cost-effective.

## Discussion

In the microsimulation model, LC + CVD screening was the most cost-effective strategy, with costs of €8274 for each 15.128 QALY, resulting in an ICUR of €8561 per QALY compared with no screening. Compared with LC screening, the extra costs were €595 for each 0.083 QALY gained, resulting in an ICUR of €7154 per QALY. Further extending screening to Big-3 resulted in limited additional health benefits (0.009 QALYs) at an additional cost (€1,208), resulting in an ICUR of €137,690 per QALY compared with LC + CVD screening. Screening for LC only, compared with no screening, resulted in incremental costs of €1342, incremental QALYs of 0.143, and an ICUR of €9378 per QALY. This ICUR value is lower than that for comparable literature, as the probability of quitting smoking after individuals participate in screening is built into the model. Smoking cessation affects the probability of developing LC; in this model, smoking cessation also affects the probability of experiencing CVD events and the probability of experiencing COPD exacerbations. All these factors affect both the costs and the effects of screening and nonscreening to different degrees. In addition, an imperfect screening participation rate was incorporated, resulting in a lower ICUR. Extending the screening to include COPD was dominated by LC + CVD screening.

These results reveal that LC + CVD screening is likely to be cost-effective compared with all alternatives (no screening and LC screening) for the entire range of WTP thresholds used in the Netherlands (€20,000–80,000 per QALY). It is also clear that the addition of emphysema screening to LDCT screening is unlikely to be cost-effective.

This model is flexible and can be easily adjusted in the future as new data become available, providing more information at the individual level regarding COPD risk and progression, coronary calcium development over time, and current costs in the Netherlands (LC, COPD treatment, and COPD exacerbations). It remains challenging to model multiple (co-occurring) diseases to capture the full impact of treatment and smoking cessation after screening for different risks to individuals because the impact of treating one disease may also affect the (risk of developing) other diseases. This greatly increases model complexity, requires substantial and detailed evidence (currently not all available at the patient level), and requires careful validation and interpretation of the impact of screening on intermediate outcomes, as these are directly connected across diseases. For example, the number of LC deaths in the model exceeded the number of CVD deaths, both with and without LC screening. Nevertheless, in the NLST results, the number of LC deaths exceeded the number of CVD deaths only in the radiography group, whereas the LDCT screening group had fewer LC deaths than CVD deaths.^5^ This means that this model could underestimate the number of CVD deaths (21.8% in the model with LC screening versus 26.1% in the NLST trial LDCT group). This discrepancy may be due to an underestimation of the CVD risk in the model and a potential overestimation of the impact of smoking cessation.

This study had certain limitations. First, and most importantly, the model had some limitations. First, the input data were not ideal. Some data are relatively old (e.g., COPD mortality hazard ratios which were used to sufficiently represent the effect of treatment on mortality through the reduction of exacerbations), some data are from countries that are not necessarily comparable to the Netherlands in terms of the health care system and subsequently the costs and quality of life, and some data are from a different target population than the NLST population, which this study focuses on. Furthermore, data were combined from different sources that sometimes used slightly different definitions, for example, what is considered a moderate or severe COPD exacerbation or what is considered CHD. In addition, some potentially relevant influences of individuals’ characteristics on their risks were not captured in this model because data to define such relationships were not available. For example, increased mortality risk in patients with emphysema and CVD has not been incorporated. The included LC data mostly reflected non-small cell LC, which is the most common histopathology. The radiation-induced risk of developing cancer owing to repeated LDCT screening was not modeled, whereas the additional 10-year risk of radiation-induced major cancers is known to be 0.05%.^56^ Finally, by using the diameter of tumors, the assumption is that all tumors are spherical, as far as growth and detection are concerned. Tumors with different shapes might result in larger or smaller tumor volumes at detection without screening than those used in this model, influencing the size, stage, and subsequent health outcomes. Although we have chosen our evidence to derive parameter values very carefully and combined them, our parameter estimates may provide a slightly positive perspective on the potential benefits of Big-3 screening. Future research will indicate whether the parameter values we currently deem realistic are in fact over- or underestimated.

In addition to the cost-effectiveness of Big-3 screening, this modeling study reported the overlapping effects of diseases with similar risk factors. This became clear with the interpretation and comparison of intermediate outcomes when screening for different disease combinations and the cost-effectiveness of LC screening compared with no screening which deviates from other published studies. This highlights not only the importance of including as many related comorbidities as possible when building health economic models but also shows that the effect of LC screening might have been underestimated in previous cost-effectiveness studies, in which the effect of smoking cessation, after participating in screening, on diseases other than LC was not (fully) taken into account.

Future research can easily use and expand this model as it is built flexibly and is open source. As new data become available from trials investigating emphysema and CVD screening, the model can be updated to provide new health and economic estimates reflecting these new data. Relevant model extensions could include the use of baseline emphysema quantification as a risk predictor for developing LC to assess the benefits of personalized LC screening frequency.

In conclusion, the current evidence suggests that extending the LC screening results in a more cost-effective screening program. The addition of CVD screening to LC screening is highly likely to be cost-effective. Further analyses using this model may provide insights into the benefits of optimizing target subpopulations to screen and optimize individual screening frequencies.

## CRediT Authorship Contribution Statement

**Carina M. Behr:** Conceptualization, Methodology, Software, Validation, Formal analysis, Investigation, Data curation, Writing - original draft, Visualization.

**Maarten J. IJzerman:** Conceptualization, Validation, Investigation, Writing - review & editing, Supervision, Funding acquisition.

**Michelle M.A. Kip:** Conceptualization, Methodology, Validation, Investigation, Writing - review & editing.

**Harry J.M. Groen:** Validation, Investigation, Writing - review & editing.

**Marjolein A. Heuvelmans:** Validation, Investigation, Writing - review & editing.

**Maarten van den Berge:** Validation, Investigation.

**Pim van der Harst:** Validation, Investigation.

**Marleen Vonder:** Validation, Investigation.

**Rozemarijn Vliegenthart:** Validation, Investigation, Writing - review & editing, Supervision, Funding acquisition.

**Hendrik Koffijberg:** Conceptualization, Methodology, Formal analysis, Investigation, Writing - review & editing, Supervision.

## Disclosure

Prof. Groen received funding for this work from the Netherlands Organisation for Health Research and Development; and payment for a presentation from Eli Lilly. Prof. van den Berge declares grants or contracts from 10.13039/100004330GlaxoSmithKline, 10.13039/100004337Roche, and Novartis. Prof. Vliegenthart declares grants or contracts from the Dutch Cancer Foundation, 10.13039/501100011699Siemens Healthineers, and the 10.13039/100002129Dutch Heart Foundation; payment or honoraria from Siemens Healthineers and Bayer Healthcare and participation; and participated in a data safety monitoring board for the Institute for Cardiometabolism and Nutrition is president of the European Society of Cardiovascular Radiology and deputy editor of Radiology. The remaining authors declare no conflict of interest.
