# Erratum to ‘Model-Based Cost-Utility Analysis of Combined Low-Dose Computed Tomography Screening for Lung Cancer, Chronic Obstructive Pulmonary Disease, and Cardiovascular Disease’ [JTO Clinical and Research Reports Volume 6 Issue 5 (2025) 100813]

**DOI:** 10.1016/j.jtocrr.2025.100841

**Published:** 2025-05-09

**Authors:** Carina M. Behr, Maarten J. IJzerman, Michelle M.A. Kip, Harry J.M. Groen, Marjolein A. Heuvelmans, Maarten van den Berge, Pim van der Harst, Marleen Vonder, Rozemarijn Vliegenthart, Hendrik Koffijberg

**Affiliations:** a Health Technology and Services Research, TechMed Centre, University of Twente, Enschede, The Netherlands; b Erasmus School of Health Policy & Management, Erasmus University Rotterdam, Rotterdam, The Netherlands; c Department of Pulmonary Diseases and Tuberculosis, UMCG - University Medical Center Groningen, Groningen, The Netherlands; d Department of Epidemiology, University Medical Center of Groningen, University of Groningen, Groningen, The Netherlands; e Department of Pulmonary Diseases, University of Groningen, University Medical Center Groningen, Groningen, The Netherlands; f Groningen Research Institute for Asthma and COPD, University of Groningen, University Medical Center Groningen, Groningen, The Netherlands; g Department of Cardiology, University Medical Center Utrecht, Utrecht, The Netherlands; h Department of Radiology, Medical Imaging Center, University Medical Center Groningen, University of Groningen, Groningen, The Netherlands; i Machine Learning Lab, Data Science Center in Health (DASH), University Medical Center Groningen, University of Groningen, Groningen, The Netherlands

In the original published version of this article, an error was introduced during the copyediting process by the Suppliers.

The text originally published “Several studies have investigated these diseases.”

Has been corrected to “Some studies investigate specific diseases."

This error bears no reflection on the article or its authors. The publisher apologizes to the authors and the readers for this unfortunate error.

