# Supplementary material for: Model-Based Cost-Utility Analysis of Combined Low-Dose Computed Tomography Screening for Lung Cancer, Chronic Obstructive Pulmonary Disease, and Cardiovascular Disease
Source: JTO Clin Res Rep. 2025 Feb 19;6(5):100813. doi: 10.1016/j.jtocrr.2025.100813 (PMC11998116; doi:10.1016/j.jtocrr.2025.100813)
Supplement: Supplementary Material [file mmc1.docx]

Supplementary material: Model based cost-utility analysis of LDCT screening for lung cancer, COPD and CVD

Table of Contents

[Survival curves of LC stages 2](#_Toc152574796)

[Calibration of warm-up time 3](#_Toc152574797)

[Deterministic results 3](#_Toc152574798)

[Stage distribution of no-screening and LC screening 4](#_Toc152574799)

[AdViSHE Checklist 4](#_Toc152574800)

[CHEERS Checklist 2022 8](#_Toc152574801)

# Survival curves of LC stages


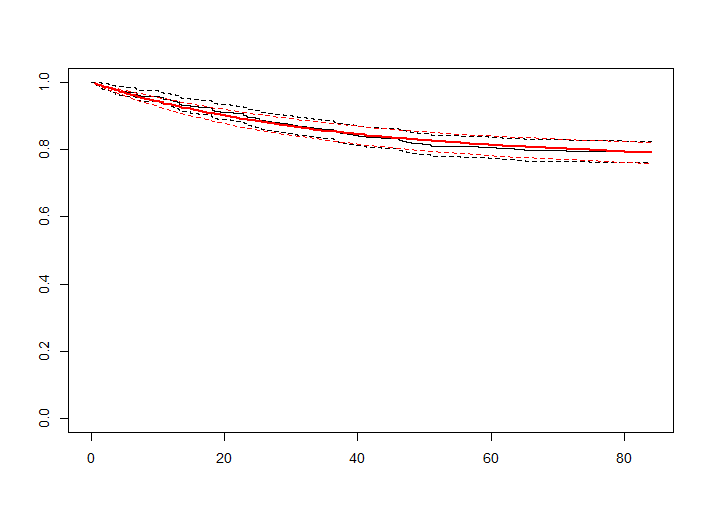

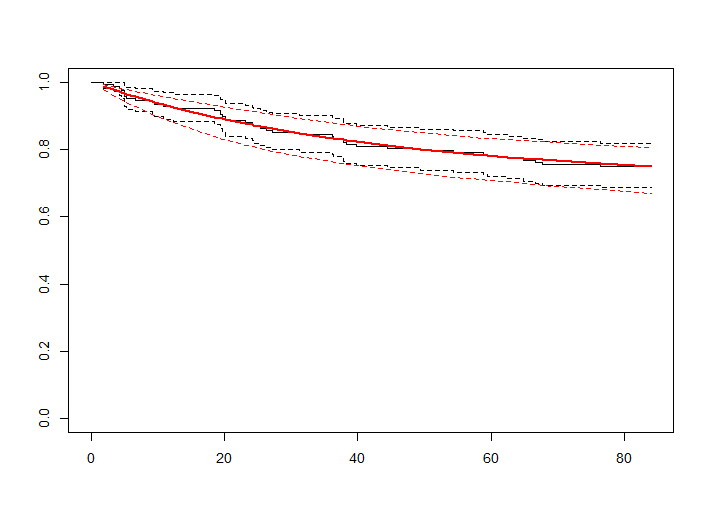

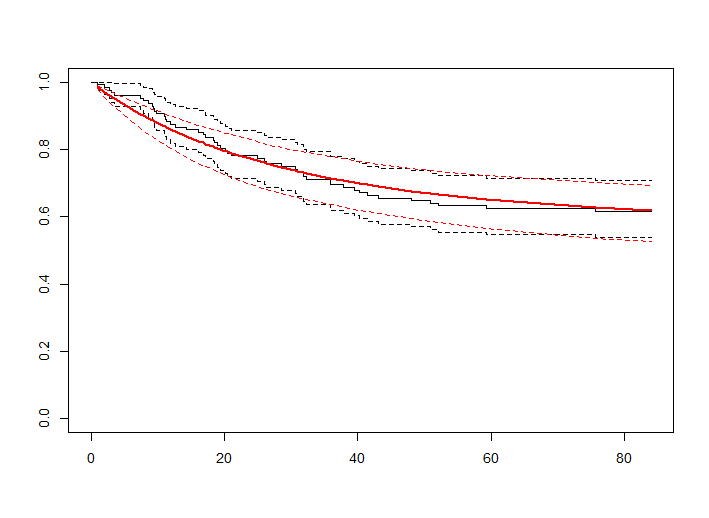

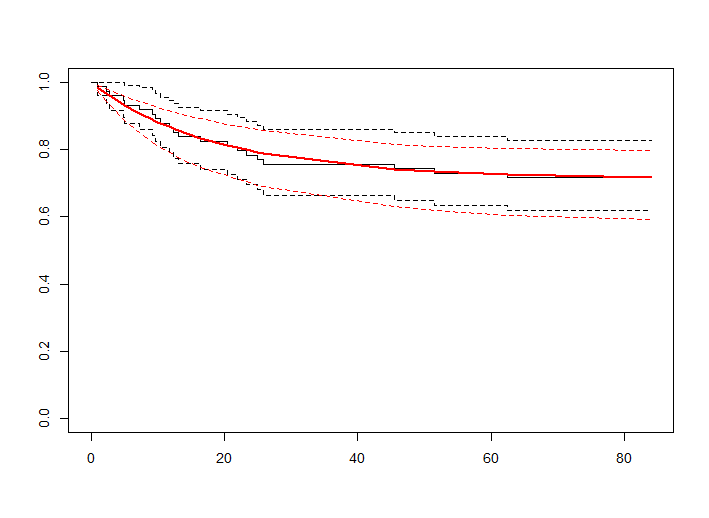

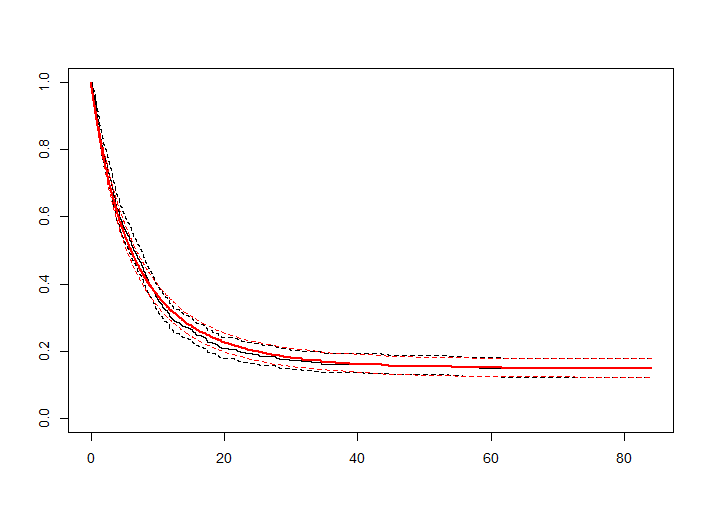

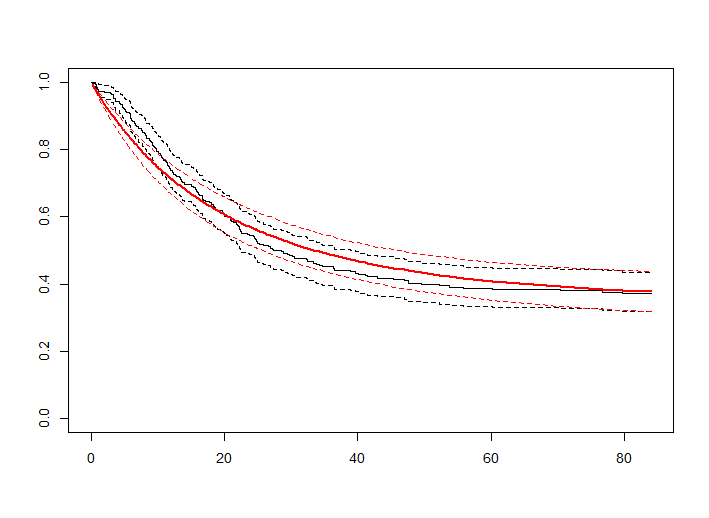

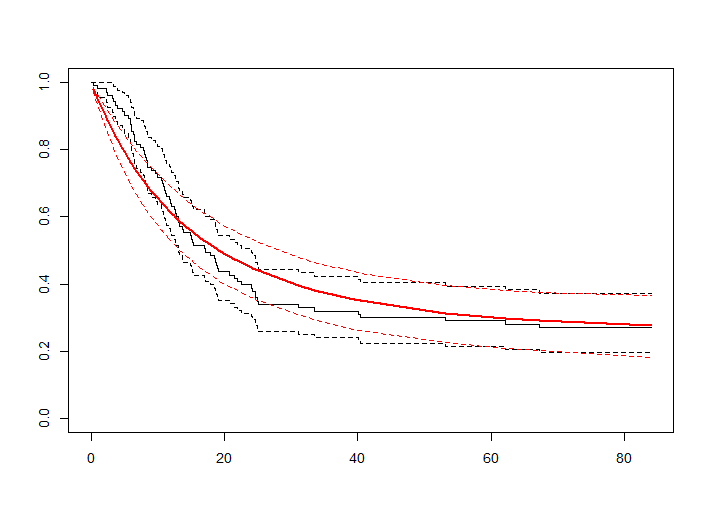


Proportion of individuals alive

Time (months)

Stage IA

Stage IB

Stage IIA

Stage IIB

Stage IIIA

Stage IIIB

Stage IV

Figure 1 Survival curves for stage IA to IV lung cancer. Black curve represents NLST patient-level data, red curve is the fitted gompertz distribution used in the simulation model with a 95% confidence interval

# Calibration of warm-up time

The calibration of warm-up time and other calibration was performed using the deterministic analysis model. The Lung cancer (LC) detection rate from NELSON after the first screening round was 0.9%. Therefore, the duration of the warm-up time as explained in the manuscript was calibrated to result in approximately 0.9% of the screening population having LC when the first screening round starts in the model. This resulted in an optimal warm-up period of 48 months, which led to 90/10,000 individuals with undetected tumours at the start of the first screening round.

# Deterministic results

Table 1 Deterministic cost-effectiveness results for screening for different combinations of diseases

| **Strategy** | **Cost** | **Effects (QALYs)** | **Incremental Costs** | **Incremental Effects** | **ICER** | **Status** |
| --- | --- | --- | --- | --- | --- | --- |
| No screening | 10,790 | 14.971 | - | - | - | ND |
| LC+CVD screening | 12,509 | 15.142 | 1,719 | 0.1720 | 9,992 | ND |
| Big-3 screening | 13,750 | 15.155 | 1,240 | 0.0124 | 100,204 | ND |
| LC screening | 12,237 | 15.086 | - | - |  | D |
| LC+COPD screening | 13,392 | 15.095 | - | - | - | D |
| ND: Not dominated; D: Dominated; | | | | | | |


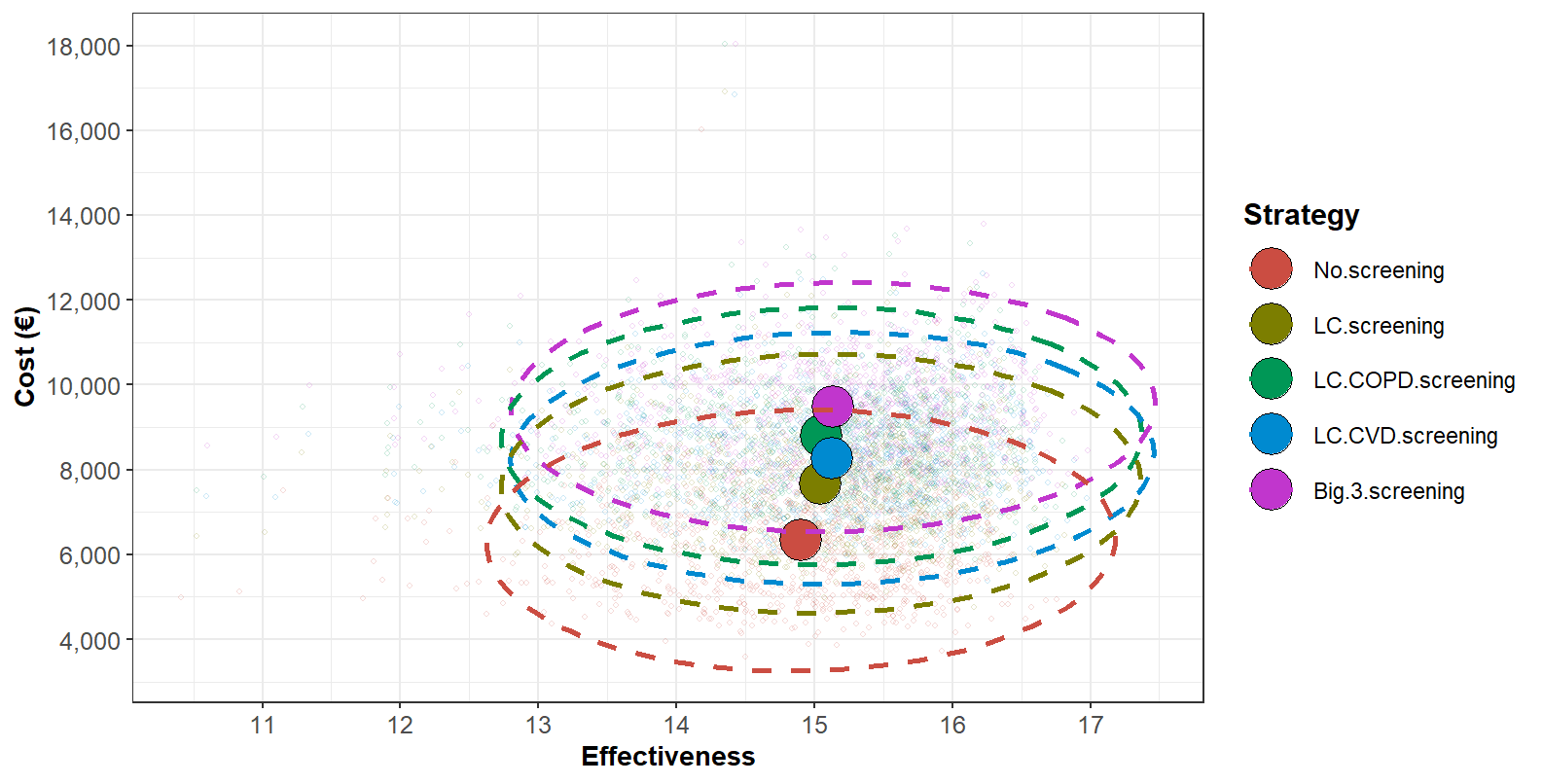


**(Quality adjusted life years)**

Figure 2 Cost-effectiveness plane with 95% confidence interval)

Figure 2 shows the absolute costs and effects of each screening strategy under uncertainty. From the graph, the additional costs and effects of both LC+CVD screening and Big-3 screening compared to No-screening and LC screening respectively can be read. The 95% confidence interval of each strategy is also shown.

**Stage distribution of no-screening and LC screening**

The deterministic model was run for no-screening and three rounds (year 1, year 3 and year 5.5) of LC screening and the stage distribution of detected lung cancers was recorded after 10-years. The NELSON LC stage distribution was recalculated with only the cases with a known stage, assuming that all cancers reported as having unknown stage were distributed similarly to the known stages.

Table 1 Stage distribution of detected lung cancers in a no-screening strategy from the model, compared to stage distributions reported in, or recalculated from, published literature

| **Stage** | **Netherlands (NSCLC)** (iknl 2012) | **NELSON** (de Koning et al. 2020) | **NELSON recalculated** | **NLST (7 years)** (Louise et al. 2011) | **NLST (10 years)** (Aberle et al. 2019) | **Model** |
| --- | --- | --- | --- | --- | --- | --- |
| I | 24% | 13,5% | 14,3% | 31,1% | 27,5% | 24,5% |
| II | 8% | 9,9% | 10,5% | 7,9% | 9,1% | 9,9% |
| III | 20% | 25,3% | 26,8% | 24,8% | 19,1% | 23,2% |
| IV | 48% | 45,7% | 48,4% | 36,1% | 35,5% | 42,4% |

Table 2 Stage distribution of detected lung cancers amongst those who participate in lung cancer screening after 10 years in which 3 rounds of screening were offered from the model, compared to stage distributions reported in, or recalculated from, published literature

| **Stage** | **NELSON** (de Koning et al. 2020) | **NELSON recalculated** | **NLST (7 years)** (Louise et al. 2011) | **NLST (10 years)** (Aberle et al. 2019) | **Model** |
| --- | --- | --- | --- | --- | --- |
| I | 40,4% | 43,3% | 50,0% | 39,6% | 54,4% |
| II | 8,4% | 9,0% | 7,1% | 8,5% | 8,8% |
| III | 17,7% | 19,0% | 21,2% | 17,5% | 16,7% |
| IV | 26,7% | 28,7% | 21,7% | 27,5% | 20,1% |

# AdViSHE Checklist

**AdViSHE Assessment of the Validation Status of Health-Economic decision models Part A: Validation of the conceptual model (2 questions) Part A discusses techniques for validating the conceptual model. A conceptual model describes the underlying system (e.g., progression of disease) using a mathematical, logical, verbal, or graphical representation. Please indicate where the conceptual model and its underlying assumptions are described and justified.**

A graphical representation of the health states constructing the Markov model was made during conceptualisation. Furthermore, lung cancer (LC) progression was modelled using volume doubling time similar to in (Du et al. 2020). The structure of the model is based on expert opinion and by investigating similar health economic evaluations from a previous systematic review (Behr et al. 2023). The underlying is assumptions are described in the manuscript accompanying this checklist.

**A1/ Face validity testing (conceptual model): Have experts been asked to judge the appropriateness of the conceptual model? If yes, please provide information on the following aspects: - Who are these experts? - What is your justification for considering them experts? - To what extent do they agree that the conceptual model is appropriate? If no, please indicate why not.**

The model was validated during development phases by experts including health economic modellers, pulmonologists, epidemiologists, a radiologist and a cardiologist who also form part of the coauthors of this paper. Three formal validation rounds focused on the model structure, the input variables and the interim as well as final outcomes

**A2/ Cross validity testing (conceptual model): Has this model been compared to other conceptual models found in the literature or clinical textbooks? If yes, please indicate where this comparison is reported. If no, please indicate why not.**

Based on published articles found in a systematic review (Behr et al. 2023), models for LC screening and calcium scoring as a form of screening were investigated. Our model structure and decisions were consequently based on a combination of the data that was available, previous models and what is reasonably possible in an individual-level health state transition model.

**Part B: Input data validation (2 questions)**

**Part B discusses techniques to validate the data serving as input in the model. These techniques are applicable to all types of models commonly used in HE modelling. Please indicate where the description and justification of the following aspects are given: - search strategy; - data sources, including descriptive statistics; - reasons for inclusion of these data sources; - reasons for exclusion of other available data sources; - assumptions that have been made to assign values to parameters for which no data was available; - distributions and parameters to represent uncertainty; - data adjustments: mathematical transformations (e.g., logarithms, squares); treatment of outliers; treatment of missing data; data synthesis (indirect treatment comparison, network meta-analysis); calibration; etc**

Model inputs were ideally used from the individual-level, Nationals Lung Screening Trial (NLST) data, furthermore, cohort level data were ideally used based on populations within the Netherlands, other similar EU countries, or from abroad if the needed data were not available from ideal sources. The populations were also ideally the NLST population, a comparable LC screening population or a group of current and former smokers. If data from these populations were not available, cardiovascular disease (CVD) or chronic obstructive pulmonary disease (COPD) patient populations or general population data was used. Data was always evaluated for quality, preferring systematic reviews and randomised control trials and confirming that the values are validated to what the expert panel expected. Due to a large individual-level databased from NLST, the missing values which were needed from specific datapoints were excluded. Rates were converted to probabilities, risks, probabilities and costs were converted to monthly values. Unless an input data source specified otherwise, or data fitted on NLST data has a different most fitting distribution, probabilities and utilities values were represented by beta distributions and costs by gamma distributions. The input data with their respective distributions are presented in the Methods section.

**B1/ Face validity testing (input data): Have experts been asked to judge the appropriateness of the input data? If yes, please provide information on the following aspects: - Who are these experts? - What is your justification for considering them experts? - To what extent do they agree that appropriate data have been used? If no, please indicate why not.**

The input parameters were discussed within the group of experts, separately for cardiology and pulmonology. The inputs used were found to be the most appropriate data available, and where the appropriateness of the data is questionable, it is mentioned in the manuscript.

**B2/ Model fit testing: When input parameters are based on regression models, have statistical tests been performed? If yes, please indicate where the description, the justification and the outcomes of these tests are reported. If no, please indicate why not.**

No unpublished regression models were used in the model. The prediction models for developing lung cancer and cardiovascular diseases are previously published, validated models. The regression models for predicting willingness to participate, were evaluated as underperforming and was therefore excluded from the final simulation model.

**Part C: Validation of the computerized model (4 questions)**

**Part C discusses various techniques for validating the model as it is implemented in a software program. If there are any differences between the conceptual model (Part A) and the final computerized model, please indicate where these differences are reported and justified.**

There are no distinct differences between the conceptual and final models, but the final model includes more detail which could not be captures in the graphical representation of the conceptual model. The model was built using the DARTH framework (Krijkamp et al. 2018) in R version 4.2.2 (R Core Team 2022).

**C1/ External review: Has the computerized model been examined by modelling experts? If yes, please provide information on the following aspects: - Who are these experts? - What is your justification for considering them experts? - Can these experts be qualified as independent? - Please indicate where the results of this review are reported, including a discussion of any unresolved issues. If no, please indicate why not.**

The model was built by a phd candidate in health economic modelling, with the assistance of two modelling experts (Professor of Technology Assessment of Digital Health Innovations and assistant professor of health technology assessment), both with experience in teaching and research applying health economic modelling. No formal review has been conducted.

**C2/ Extreme value testing: Has the model been run for specific, extreme sets of parameter values in order to detect any coding errors? If yes, please indicate where these tests and their outcomes are reported. If no, please indicate why not.**

The model was run with extreme sets of input parameters to detect coding errors. Tests included setting costs or effects to zero and not allowing transitions. The test results were not reported in the manuscript as this was for model validation purposes only and no errors were found.

**C3/ Testing of traces: Have patients been tracked through the model to determine whether its logic is correct? If yes, please indicate where these tests and their outcomes are reported. If no, please indicate why not.**

The patient trace matrix was used to checked how the total number of individuals in each health state over time as well as on individual level. The costs and effects were checked over time on cohort level. The test results were not included in the manuscript, as no errors were found.

**C4/ Unit testing: Have individual sub-modules of the computerized model been tested? If yes, please provide information on the following aspects: - Was a protocol that describes the tests, criteria, and acceptance norms defined beforehand? - Please indicate where these tests and their outcomes are reported. If no, please indicate why not.**

Sub-modules were tested by checking the inputs and outputs of the function during a model run, however this was not tested systematically and therefore, the results of these tests are also not reported.

**Part D: Operational validation (4 questions)**

**Part D discusses techniques used to validate the model outcomes. We validated the model outcomes in terms of face validation (group discussion), cross-validation (comparing to another model), and using alternative input data.**

**D1/ Face validity testing (model outcomes): Have experts been asked to judge the appropriateness of the model outcomes? If yes, please provide information on the following aspects: - Who are these experts? - What is your justification for considering them experts? - To what extent did they conclude that the model outcomes are reasonable? If no, please indicate why not**.

The model outcomes were validated during development phases by experts including health economic modellers, pulmonologists, epidemiologists, a radiologist and a cardiologist who also form part of the coauthors of this paper. Three formal validation rounds focused on the model structure, the input variables and the interim as well as final outcomes. Model outcomes were found to be partially reasonable, with problems highlighted in the article including a discussion on the extension of the model when more appropriate data becomes available

**D2/ Cross validation testing (model outcomes): Have the model outcomes been compared to the outcomes of other models that address similar problems? If yes, please provide information on the following aspects: - Are these comparisons based on published outcomes only, or did you have access to the alternative model? - Can the differences in outcomes between your model and other models be explained? - Please indicate where this comparison is reported, including a discussion of the comparability with your model. If no, please indicate why not.**

The model outcomes were compared to those found for other LC screening models, however comparison was difficult, as this model includes a wider range of events and disutilties caused by three diseases. This issue was raised and discussed in the manuscript.

**D3/ Validation against outcomes using alternative input data: Have the model outcomes been compared to the outcomes obtained when using alternative input data? If yes, please indicate where these tests and their outcomes are reported. If no, please indicate why not.**

We applied different values of the positive predictive value of the pre-hospital scale in scenario analysis. The total costs, and quality-adjusted life years were reported accordingly in the manuscript. In addition, we ran the model with alternative input parameters of transition probabilities of mRS from three months to one year to check the outcomes (eTable 8). These transition probabilities were derived from the UK cohort study.9 We reported the results in the AdViSHE section but not in the manuscript as we preferred input data from the Netherlands. The DTAS strategy was still cost-effective when applying alternative transition probabilities from the UK. eTable 8 Cost-effectiveness results when applying transition probabilities from the UK Strategy Cost per patient ($) Incremental cost per patient ($) QALY per patient Incremental QALY per patient ICER ($/QALY) Base case ITER 92,634 - 2.42 - - DTAS 108,723 16,089 3.06 0.65 24,925 Applying transition probabilities from the UK ITER 88,156 - 2.47 - - DTAS 101,289 13,133 3.13 0.66 19,898

**D4/ Validation against empirical data: Have the model outcomes been compared to empirical data? If yes, please provide information on the following aspects: - Are these comparisons based on summary statistics, or patient-level datasets? - Have you been able to explain any difference between the model outcomes and empirical data? - Please indicate where this comparison is reported. If no, please indicate why not.**

Not applicable as we used input data from different randomized controlled trials (the ANGIOCAT trial for short-term outcomes and transition probabilities from the MR CLEAN trial for long-term outcomes) and other resources (i.e., Dutch mortality rate).

**Part E: Other validation techniques (1 question) E1/ Other validation techniques: Have any other validation techniques been performed? If yes, indicate where the application and outcomes are reported, or else provide a short summary here.**

Not applicable

# CHEERS Checklist 2022

(Husereau et al. 2022)

| **Topic** | **No.** | **Item** | **Location where item is reported** |
| --- | --- | --- | --- |
| **Title** |  |  |  |
|  | 1 | Identify the study as an economic evaluation and specify the interventions being compared. | Title, Page 2 |
| **Abstract** |  |  |  |
|  | 2 | Provide a structured summary that highlights context, key methods, results, and alternative analyses. | Abstract, Page 2 |
| **Introduction** |  |  |  |
| **Background and objectives** | 3 | Give the context for the study, the study question, and its practical relevance for decision making in policy or practice. | Introduction, Line 72-74 |
| **Methods** |  |  |  |
| **Health economic analysis plan** | 4 | Indicate whether a health economic analysis plan was developed and where available. | No plan was set up, as the project started before the newest version of the CHEERS checklist |
| **Study population** | 5 | Describe characteristics of the study population (such as age range, demographics, socioeconomic, or clinical characteristics). | Methods, Line 90-91 |
| **Setting and location** | 6 | Provide relevant contextual information that may influence findings. | Methods, Lines 82-83 |
| **Comparators** | 7 | Describe the interventions or strategies being compared and why chosen. | Methods, Lines 76-78 |
| **Perspective** | 8 | State the perspective(s) adopted by the study and why chosen. | Methods, Lines 78 |
| **Time horizon** | 9 | State the time horizon for the study and why appropriate. | Methods, Lines 78 |
| **Discount rate** | 10 | Report the discount rate(s) and reason chosen. | Methods, Lines 78 |
| **Selection of outcomes** | 11 | Describe what outcomes were used as the measure(s) of benefit(s) and harm(s). | Methods, Lines 147-148 |
| **Measurement of outcomes** | 12 | Describe how outcomes used to capture benefit(s) and harm(s) were measured. | Not reported, the measurement of quality of life depends on available data for every application. All input parameters are described in Table 1. |
| **Valuation of outcomes** | 13 | Describe the population and methods used to measure and value outcomes. | Methods, Table 1. |
| **Measurement and valuation of resources and costs** | 14 | Describe how costs were valued. | Not reported, the measurement of costs depend on available data for every application. All input parameters are described in Table 1. |
| **Currency, price date, and conversion** | 15 | Report the dates of the estimated resource quantities and unit costs, plus the currency and year of conversion. | Methods, Table 1 |
| **Rationale and description of model** | 16 | If modelling is used, describe in detail and why used. Report if the model is publicly available and where it can be accessed. | Methods, Paragraphs 1-3 |
| **Analytics and assumptions** | 17 | Describe any methods for analysing or statistically transforming data, any extrapolation methods, and approaches for validating any model used. | Methods , Subheading Analysis and Appendix |
| **Characterising heterogeneity** | 18 | Describe any methods used for estimating how the results of the study vary for subgroups. | Not reported, as subgroups and their respective sizes are incorporated by building a microsimulation model. |
| **Characterising distributional effects** | 19 | Describe how impacts are distributed across different individuals or adjustments made to reflect priority populations. | Not reported, as subgroups and their respective sizes are incorporated by building a microsimulation model. |
| **Characterising uncertainty** | 20 | Describe methods to characterise any sources of uncertainty in the analysis. | Methods under the heading Analysis |
| **Approach to engagement with patients and others affected by the study** | 21 | Describe any approaches to engage patients or service recipients, the general public, communities, or stakeholders (such as clinicians or payers) in the design of the study. | Methods under Validation |
| **Results** |  |  |  |
| **Study parameters** | 22 | Report all analytic inputs (such as values, ranges, references) including uncertainty or distributional assumptions. | Methods, Table 1 |
| **Summary of main results** | 23 | Report the mean values for the main categories of costs and outcomes of interest and summarise them in the most appropriate overall measure. | Results, first paragraph |
| **Effect of uncertainty** | 24 | Describe how uncertainty about analytic judgments, inputs, or projections affect findings. Report the effect of choice of discount rate and time horizon, if applicable. | Results, under subheading sensitivity analysis |
| **Effect of engagement with patients and others affected by the study** | 25 | Report on any difference patient/service recipient, general public, community, or stakeholder involvement made to the approach or findings of the study | Not reported |
| **Discussion** |  |  |  |
| **Study findings, limitations, generalisability, and current knowledge** | 26 | Report key findings, limitations, ethical or equity considerations not captured, and how these could affect patients, policy, or practice. | Discussion |
| **Other relevant information** |  |  |  |
| **Source of funding** | 27 | Describe how the study was funded and any role of the funder in the identification, design, conduct, and reporting of the analysis | End of manuscript |
| **Conflicts of interest** | 28 | Report authors conflicts of interest according to journal or International Committee of Medical Journal Editors requirements. | End of manuscript |

# References

Aberle, Denise R. et al. 2019. “Lung Cancer Incidence and Mortality with Extended Follow-up in the National Lung Screening Trial.” *Journal of Thoracic Oncology* 14(10): 1732–42. https://linkinghub.elsevier.com/retrieve/pii/S1556086419304733.

Behr, Carina M et al. 2023. “Population-Based Screening Using Low-Dose Chest Computed Tomography: A Systematic Review of Health Economic Evaluations.” *PharmacoEconomics* 41(4): 395–411. https://doi.org/10.1007/s40273-022-01238-3.

Du, Yihui et al. 2020. “Cost-Effectiveness of Lung Cancer Screening with Low-Dose Computed Tomography in Heavy Smokers: A Microsimulation Modelling Study.” *European Journal of Cancer* 135: 121–29. http://www.sciencedirect.com/science/article/pii/S0959804920302653 (January 25, 2021).

Husereau, Don et al. 2022. “Consolidated Health Economic Evaluation Reporting Standards (CHEERS) 2022 Explanation and Elaboration: A Report of the ISPOR CHEERS II Good Practices Task Force.” *Value in Health* 25(1): 10–31. https://doi.org/10.1016/j.jval.2021.10.008.

iknl. 2012. “Overleving | Longcarcinoom; Stadium.” http://www.cijfersoverkanker.nl/selecties/dataset_3/img59a2f3e0a1c40.

de Koning, Harry J et al. 2020. “Reduced Lung-Cancer Mortality with Volume CT Screening in a Randomized Trial.” *New England Journal of Medicine* 382(6): 503–13. https://doi.org/10.1056/NEJMoa1911793 (January 12, 2022).

Krijkamp, Eline M. et al. 2018. “Microsimulation Modeling for Health Decision Sciences Using R: A Tutorial.” *Medical Decision Making* 38(3): 400–422. http://journals.sagepub.com/doi/10.1177/0272989X18754513.

Louise, C et al. 2011. “Reduced Lung-Cancer Mortality with Low-Dose Computed Tomographic Screening.” *The New England Journal of Medicine* 365(5): 395–409. https://doi.org/10.1056/NEJMoa1102873.

R Core Team. 2022. “R: A Language and Environment for Statistical Computing.” https://www.r-project.org/.
